# *Corvidae* Feather Pulp and West Nile Virus Detection

**DOI:** 10.3201/eid1005.030825

**Published:** 2004-05

**Authors:** Douglas E. Docherty, Renee Romaine Long, Kathryn M. Griffin, Emi K. Saito

**Affiliations:** * National Wildlife Health Center, Madison, Wisconsin, USA

**Keywords:** West Nile virus, feather pulp, surveillance

## Abstract

We evaluated cloacal swab, vascular pulp of flight feather, and kidney and spleen pool samples from carcasses of members of the family *Corvidae* as sources of West Nile virus (WNV). The cloacal swab, kidney and spleen pool, and feather pulp, respectively, were the source of WNV in 38%, 43%, and 77% of the carcasses.

Samples from carcasses of the family *Corvidae* have been used in the surveillance for West Nile virus (WNV) since the virus was detected in the United States in 1999 (*1*). Various laboratories have used organs such as brain, kidney, and spleen to isolate the virus in cell culture, or to detect the virus by using a variety of techniques, or both (*2*). WNV surveillance efforts (*3*,*4*) have reported 50%–70% of corvids as WNV positive. Postmortem oral and cloacal swabs, along with the brain, are suitable for detecting the virus in experimentally infected birds (*5*). Previous studies have shown that WNV may be isolated from feather pulp of experimentally infected crows (National Wildlife Health Center, unpub. data). We describe the results of WNV isolation attempts from cloacal swabs, kidney and spleen pools, and the flight feathers containing vascular pulp (*6*) of dead American Crows (*Corvus* *brachyrhynchos*) and Blue Jays (*Cyanocitta* *cristata*) that were found in the field and suspected of being WNV infected.

## The Study

Specimens were obtained at necropsy from a group of 28 American Crow and 56 Blue Jay carcasses. These birds were submitted, from August through October, as field cases in the course of the larger 2002 wildlife surveillance effort for WNV at the U.S. Geological Survey (USGS), National Wildlife Health Center, in Madison, Wisconsin. Birds received for WNV surveillance were evaluated for WNV but not for cause of death. Kidney and spleen pools, cloacal swab, and feather pulp were collected from each of these 84 birds received from the following nine states: Alabama, Illinois, Kansas, Maryland, Missouri, North Dakota, Pennsylvania, Texas, and Virginia. The birds in this study were obtained after WNV was initially detected in birds from each state; only those carcasses judged to be fresh were sampled.

At necropsy each bird was examined for wing flight feathers (remiges) and tail flight feathers (retrices) that contained vascular pulp. These feathers were pulled from the feather follicle and aseptically cut at the distal end of the umbilicus (*6*). The umbilicus, containing vascular pulp, was placed in viral transport media (*7*). At least one and up to three feathers were collected from American Crows and at least four and up to six were collected from Blue Jays. Also at necropsy, cloacal samples were taken with Dacron swabs that were swirled in viral transport media, squeezed out against the side of the collection tube, and then discarded. Kidney and spleen samples were aseptically collected at necropsy. Samples were kept chilled at 4°C, and any not processed within 24 hours after they were obtained were stored at –80°C.

In the laboratory, a 10% (wt/volume) kidney and spleen pool suspension was prepared in viral transport media. The kidney and spleen pool suspension was blended in a Stomacher 400 Circulator (Seward, Norfolk, UK) until it appeared homogeneous. The vascular pulp was aseptically removed from the umbilicus with forceps, and viral transport media was added to the available feather pulp mass to produce a 10% (wt/volume) suspension. The cloacal swab and feather pulp suspensions were vortexed until they appeared homogeneous. The kidney and spleen pool, cloacal swab, and feather pulp suspensions were centrifuged at 800 x *g* for 30 min at 4°C, and 1 mL of the supernatant was injected onto an established Vero (ATCC CRL-1587) cell monolayer in 12 cm^2^ (culture surface area) bottles. These bottles were incubated at 37°C and 2% CO_2_ and read periodically over 7 days for viral cytopathic effect (CPE). To screen for WNV, cell culture bottles showing viral CPE involving at least 75% of the Vero monolayer were harvested after one freeze-and-thaw cycle and tested for WNV by reverse transcriptase–polymerase chain reaction (RT-PCR) (*8*). The RT-PCR test was also used to determine whether feather pulp or cloacal swab suspensions, negative for virus isolation, contained quantities of WNV below the detection level of cell culture. The number of WNV plaque forming units (PFU) was determined (*9*) to evaluate the quantity of virus in various kidney and spleen pool, cloacal swab, and feather pulp samples.

With the screening method described here, WNV was isolated from 65 (77%) of 84 corvids. Of the 65 WNV-positive birds, WNV was isolated from 100% (65/65) of the feather pulp samples, 55% (36/65) of the kidney and spleen pool samples, and 49% (32/65) of the cloacal swabs. WNV was isolated from all three samples for 25% (21/84) of all birds tested. Attempts at virus isolation were significantly (p < 0.001) more successful from feather pulp than from either kidney and spleen pool or cloacal swab. The ability to successfully isolate WNV from either the kidney and spleen pool or the cloacal swab was essentially the same (p > 0.5).

The feather pulp or cloacal swab samples from birds from which WNV was not isolated were also negative for WNV by using RT-PCR. Comparisons of the quantity of WNV in 0.1 mL of sample suspension indicated that more PFU of WNV were in the feather pulp than cloacal swab or kidney and spleen pool suspensions (Table). A comparison of the number of PFU in the feather pulp samples and cloacal swabs from the same 12 birds showed that the feather pulp had significantly (p < 0.0005) more.

**Table:** Logarithmic titers of West Nile virus infectious particles (per 0.1 mL of 10% tissue suspension) present in each type of sample, as detected by plaque assay in Vero cells

Type of sample	No. tested	Median (range)
Kidney/spleen pool	7	1.0 (<1.0 to 3.3)
Cloacal swab	12	1.9 (<1.0 to 4.0)
Vascular pulp of flight feather	12	4.9 (3.5 to >7.4)

## Conclusions

WNV isolation from feather pulp is a relatively sensitive assay for surveillance of corvid carcasses. Of the 84 tested, 77% (65/84) of the birds were WNV positive by feather pulp alone, 43% (36/84) were positive by kidney and spleen pool alone, and 38% (32/84) were positive by cloacal swab alone. On the basis of our determination of the WNV titer in feather pulp, cloacal swab, and kidney and spleen pool, these results could be explained by the fact that much more virus was present in the feather pulp suspension. The 23% (19/84) negative birds consisted of 16 Blue Jays and 3 American Crows, representing birds that may have died of causes other than WNV infection. Other causes may include other infectious agents, toxins, or trauma not related to concurrent WNV infection.

Previous studies of Eastern equine encephalitis virus in Ring-necked Pheasants (*Phasianus colchicus*) and avian leucosis virus in domestic chickens (*Gallus gallus*) found virus in feather pulp up to 7 days beyond detection in blood (*10*–*12*). The virus titer in feather pulp was also much greater than in blood or cloacal swab. Avian leukosis virus could be detected in feather pulp even after antibody was detected. In a recent publication (*13*), RT-PCR was used to detect WNV in the “skin including feather tips” of goslings experimentally infected with the virus. The authors of that publication concluded that blood and skin containing feather tips could, through cannibalism, horizontally transmit sufficient virus to directly infect contact control goslings.

The duration and timing of the molt is a limiting factor in using the feather pulp sample. In much of the United States, the American Crow molt will occur from July through September, the Blue Jay from June through October, and the Common Raven (*Corvus corax*) from May through October (*14*). A feather pulp sample from corvids in the United States will therefore be available during the height of the WNV season. We recommend collecting and testing the feather pulp, considering the apparent high rate of success in detecting WNV.

The feather pulp sample is nonlethal and could be taken from birds trapped live, sampled, and released. Sufficient WNV appears to be available in samples obtained from corvid carcasses suspected to be WNV infected to infect cell culture. since none of the negative cloacal swab or feather pulp samples were positive by RT-PCR. However, for subclinical infections or from other species of birds, additional testing may be necessary to determine whether the amount of virus available in feather pulp will be sufficient for detection by virus isolation or RT-PCR.
